# Impact of the Reduction Time-Dependent Electrical Conductivity of Graphene Nanoplatelet-Coated Aligned *Bombyx mori* Silk Scaffolds on Electrically Stimulated Axonal Growth

**DOI:** 10.1021/acsabm.4c00052

**Published:** 2024-03-19

**Authors:** Jitu Mani Das, Jnanendra Upadhyay, Michael G. Monaghan, Rajiv Borah

**Affiliations:** † Life Sciences Division, Institute of Advanced Study in Science & Technology, Guwahati 781035, India; ‡ Department of Physics, Dakshin Kamrup College, Kamrup, Mirza, Assam 781125, India; § Department of Mechanical, Manufacturing and Biomedical Engineering, Trinity College Dublin, Dublin D2, Ireland; ∥ Advanced Materials and BioEngineering Research (AMBER), Centre at Trinity College Dublin and the Royal College of Surgeons in Ireland, Dublin D2, Ireland; ⊥ Trinity Centre for Biomedical Engineering, Trinity College Dublin, Dublin D2, Ireland; # CÚRAM, Centre for Research in Medical Devices, National University of Ireland, Galway H91 W2TY, Ireland

**Keywords:** silk, graphene, electroconductive biomaterials, electrical stimulation, nerve regeneration

## Abstract

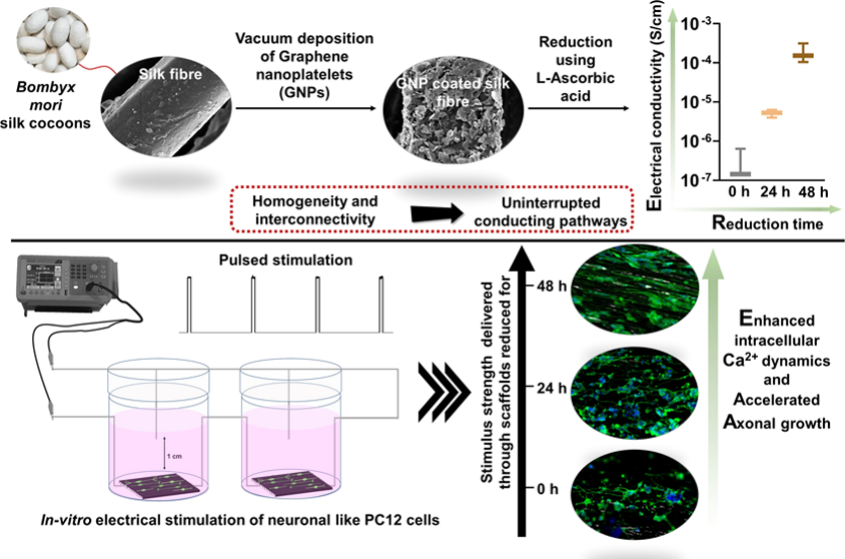

Graphene-based nanomaterials, renowned for their outstanding electrical conductivity, have been extensively studied as electroconductive biomaterials (ECBs) for electrically stimulated tissue regeneration. However, using eco-friendly reducing agents like l-ascorbic acid (l-Aa) can result in lower conductive properties in these ECBs, limiting their full potential for smooth charge transfer in living tissues. Moreover, creating a flexible biomaterial scaffold using these materials that accurately mimics a specific tissue microarchitecture, such as nerves, poses additional challenges. To address these issues, this study developed a microfibrous scaffold of *Bombyx mori* (Bm) silk fibroin uniformly coated with graphene nanoplatelets (GNPs) through a vacuum coating method. The scaffold’s electrical conductivity was optimized by varying the reduction period using l-Aa. The research systematically investigated how different reduction periods impact scaffold properties, focusing on electrical conductivity and its significance on electrically stimulated axonal growth in PC12 cells. Results showed that a 48 h reduction significantly increased surface electrical conductivity by 100–1000 times compared to a shorter or no reduction process. l-Aa contributed to stabilizing the reduced GNPs, demonstrated by a slow degradation profile and sustained conductivity even after 60 days in a proteolytic environment. β (III) tubulin immunostaining of PC12 cells on varied silk:GNP scaffolds under pulsed electrical stimulation (ES, 50 Hz frequency, 1 ms pulse width, and amplitudes of 100 and 300 mV/cm) demonstrates accelerated axonal growth on scaffolds exhibiting higher conductivity. This is supported by upregulated intracellular Ca^2+^ dynamics immediately after ES on the scaffolds with higher conductivity, subjected to a prolonged reduction period. The study showcases a sustainable reduction approach using l-Aa in combination with natural Bm silk fibroin to create a highly conductive, mechanically robust, and stable silk:GNP-based aligned fibrous scaffold. These scaffolds hold promise for functional regeneration in electrically excitable tissues such as nerves, cardiac tissue, and muscles.

## 1 Introduction

Electroconductive biomaterials (ECBs) have demonstrated superior effectiveness in the regeneration of electrically excitable tissues such as nerves, cardiac tissue, and muscles, when compared to nonconductive passive biomaterials.^1^ Notably, when combined with electrical stimulation (ES), ECBs can enhance the process of nerve regeneration.^2^ ES has been shown to increase the expression of neurotrophic factors and their receptors, leading to the upregulation of various proteins associated with regeneration, including actin, tubulin, galectin-1, growth-associated protein-43 (GAP-43), and neurotrophin-4/5 (NT-4/5), leading to faster axonal regeneration.^3^ It has been demonstrated that ES promotes axonal outgrowth by elevating intracellular neuronal cyclic adenosine monophosphate (cAMP), which subsequently activates protein kinase A (PKA) to promote the transcription of regeneration-associated proteins.^4^ To effectively enhance ES-mediated tissue regeneration, the intrinsic conductive properties of ECBs have a significant impact. ECBs need sufficient conductivity to allow for a smooth transfer of electrical charges to the cell’s outer membrane within a low range of stimulation potential that remains within safe limits for living tissues. This transfer of charges is crucial for triggering an action potential and depolarizing the membrane during ES, a process essential for the regeneration of nerve cell projections known as neurites.^5^

In this regard, a range of polymeric conductive materials, such as conducting polymers, carbon-based nanomaterials, etc., have been explored in the fabrication of flexible ECBs in various formats mimicking the target tissue microarchitectures.^6−8^ Among all of the polymeric conductive materials, graphene-based nanomaterials, including graphene, graphene oxide (GO), and reduced graphene oxide (rGO), possess excellent electrical conductivity due to their unique structure and strong C=C bonding.^9^ The high electrical conductivity of single-layer graphene is a result of its minimal defect density within the crystal lattice. Graphene is a two-dimensional (2D) nanomaterial composed of sp^2^ carbon atoms arranged in a honeycomb crystal lattice, where each atom is connected to three other carbon atoms, and one electron is freely available for electronic conduction. These free electrons presenting above and below the graphene sheet are called π electrons, providing a delocalization feature to serve as a mobile charge carrier.

Nevertheless, selecting the right synthesis method for graphene-based nanomaterials poses challenges in maintaining both their biocompatibility and conductivity simultaneously. The cost-effective, large-scale chemical synthesis of these materials frequently results in significant aggregation or restacking of graphene layers due to the strong π–π stacking interaction or van der Waals forces between them.^10^ This ultimately constrains their renowned electrical conductivity characteristics because of irreversible aggregation and inadequate ion transport. This limitation can be addressed by employing suitable reducing and capping agents. In many highly effective chemical reduction approaches, hydrazine or hydrazine hydrate is employed as the reducing agent. However, their high toxicity and volatile nature restrict their application in biological contexts. Hence, in the pursuit of a more environmentally friendly and sustainable approach, l-ascorbic acid (l-Aa) has been proven to be a safer alternative as a reducing agent.^10−12^ Nonetheless, there are difficulties in harnessing these materials to create a resilient biomaterial scaffold, especially one that replicates the microarchitecture of a specific tissue such as nerve tissue. Therefore, these materials are blended with various flexible natural and synthetic polymers such as bacterial cellulose,^13^ chitosan,^14^ collagen, gelatin,^15^ silk,^16,17^ polylactic acid (PLLA),^18^ polycaprolactone (PCL),^15^ poly(lactic-*co*-glycolic acid) (PLGA),^15^ and others. This blending is performed to create biomaterial scaffolds that exhibit characteristics such as nanofiber, porous, or tubular morphologies.^15,19^

*Bombyx mori* (Bm) silk fibroin stands out as a particularly fascinating protein-based biomaterial due to its exceptional mechanical properties compared to conventional biomaterials like collagen, gelatin, or PLLA in addition to its well-established biocompatible and tunable biodegradability.^20,21^ Bm silk fibroin has received FDA approval as a biomaterial owing to its outstanding biocompatibility, nonimmunogenicity, and adjustable biodegradability.^22^

Past studies highlighted the benefits and challenges of using graphene-based materials in conjunction with Bm silk in the form of a blend or as an external coating to evaluate their capacity to facilitate the growth of excitable tissues, such as nerve, cardiac, etc.^23^ While introducing graphene-based materials into silk fibroin, it is essential to ensure that intrinsic biocompatibility, bioactivity, and mechanical strength/flexibility of silk fibroin are not compromised. On the other hand, the electroactivity of these graphene-based materials is dependent on the reduction method as well as how the scaffold is obtained through blending or dispersion with silk fibroin or external thin coating over silk-based platforms. In the first scenario, the graphene-based materials should be homogeneously distributed in the blend or dispersion to achieve the percolation threshold so that these conductive fillers can form a well-connected conductive network within the silk fibroin matrix. In addition, achieving a high degree of reduction is limited, as access of the reducing agent to the graphene-based materials entrapped in the interior of a solid scaffold is hindered. For instance, Jafari et al. used AgNO_3_ for reduction of graphene oxide (GO) followed by raffinose grafting and then blended it with silk fibroin to fabricate a porous scaffold, which is highly resistive in nature (of the order of 10^8^ Ω) and tested with for PC12 cell proliferation.^24^ In another recent study, Magaz et al. systematically demonstrated an enhanced neurite growth on 1% (w/v) ascorbic acid-reduced graphene oxide/silk (rGO/silk)-blended fibrous scaffolds when compared to that on the GO/silk-based scaffolds.^17^ They showed a significant increase in the electrical conductivity of the rGO/silk scaffold up to 4 × 10^–5^ S/cm, leading to differences in surface roughness or protein adsorption, which ultimately contributed toward enhanced neurite growth. In contrast to the blended form as discussed above, a uniform coating of graphene-based materials over a silk-based platform is comparatively easier to achieve and could offer superior electroactivity. Nonreduced forms of graphene-based materials, such as GO, would offer interfacial interactions with the amide groups of silk fibroin for necessary adhesion, owing to the presence of oxygen-containing functional groups. For example, Zhao et al. coated GO over the electrospun silk fibers using the casting method and then reduced the GO/silk fibrous mat by immersion in 1% (w/v) ascorbic acid at 95 °C for 1 h.^25^ The electroactivity of the resultant rGO/silk scaffolds was tuned by manipulating the rGO thickness (surface resistance of the order of 10^3^ Ω). The study finally reported enhanced gap junction formation among cardiomyocytes grown on the thicker rGO/silk scaffolds under ES. In a very recent study, the same group showed the efficacy of the highly conductive rGO/silk scaffold developed using their previous approach in restoration of electrical coupling following a myocardial infarction in rat models.^26^ Aznar-Cervantes et al. also coated the electrospun silk fibroin mats with GO followed by ascorbic acid reduction and demonstrated improved neural differentiation of PC12 cells under ES.^27^

Nonetheless, the effect of ascorbic acid reduction time on the electroconductivity of silk:graphene-based materials and its correlation with electrically stimulated tissue regeneration are still unknown. A concern associated with the external coating of graphene materials over silk fibroin is the decrease in the interfacial strength (due to the removal of polar groups present in graphene materials) of the scaffold postreduction process, which can potentially result in their delamination and ultimately the conductivity stability under long-term physiological condition. Hence, an optimized reduction protocol as well as long-term stability needs to be explored.

Aligned with the previously mentioned concept, our hypothesis revolves around leveraging the interfacial interaction between the polar functional groups found in Bm silk fibroin and GNPs. This interaction aims to achieve a consistent coating of GNPs across the Bm fibers, allowing for an optimal reduction protocol to produce a highly conductive Bm:GNP-based electroconductive biomaterial.

The present study reports the fabrication of an aligned fibrous scaffold using degummed Bm fibers cross-linked with 1 wt % Bm silk fibroin. The degummed Bm fibrous scaffold was coated with graphene nanoplatelets (GNPs) through a vacuum coating process at 60 °C. The GNP-coated aligned Bm scaffold, i.e., Bm:GNP, was treated with ascorbic acid to remove oxygen-containing functional groups (e.g., –OH, –COOH) in GNPs for two different time points to assess its impact on the electrical conductivity of the scaffolds (i.e., Bm:rGNP-24 h and Bm:rGNP-48 h). The consequent impact of the reduction of GNPs has finally been assessed in terms of neural differentiation of PC12 cells and neurite growth under pulsed electrical stimulation (ES) using different variations of aligned Bm scaffolds. The intracellular Ca^2+^ level was also monitored immediately after the ES to understand the possible regulating mechanism of electrically stimulated axonal growth.

## 2 Materials and Methods

### 2.1 Materials

*Bombyx mori* silk cocoons were sourced from Shri Harihareshwara Agro Foods Industries, Karnataka, India. Sodium carbonate (Na_2_CO_3_), lithium bromide (LiBr), graphene nanoplatelet (GNP) powder (a low dimensional carbon nanoplatelet with a lateral dimension of about 2–3 μ and a few layers thick), and l-ascorbic acid (l-Aa) were procured from Sigma-Aldrich and utilized for material fabrication. Cell culture reagents such as Gibco Ham’s F-12 Nutrient Mix, fetal bovine serum (FBS), horse serum (New Zealand origin), and penicillin–streptomycin (10,000 U/mL) were received from Invitrogen, whereas nerve growth factor β (NGF-β) from rats was purchased from Sigma-Aldrich. The primary rabbit polyclonal anti-b III tubulin antibody (ab18207) and the secondary goat antirabbit IgG H&L (Alexa Fluor 488) antibody (ab150077) were obtained from Abcam, which were used for immunostaining of PC12 cells. 4,6-Diamidino-2-phenylindole (DAPI) (D3571) and Fluo-4, AM (F14201) were procured from Invitrogen.

### 2.2 Degumming of *Bombyx mori* Silk Fibers and Extraction of Silk Fibroin

*Bombyx mori* (Bm) fibers were acquired through the degumming process of cocoons, and subsequently, silk fibroin was isolated from these processed fibers following previously published protocol, with slight optimization depending upon the raw silk quality.^20,28−30^ Briefly, the cocoons were first sliced into smaller segments and then subjected to a degumming process by boiling them in a 0.02 M Na_2_CO_3_ solution for 15–20 min. Afterward, the resulting silk fibers underwent thorough rinsing with deionized water and dried overnight. Subsequently, the fibers were digested in a 9.3 M LiBr solution at 60 °C for 3–4 h. Typically, 5 g of degummed silk fibers were dissolved in a 9.3 M LiBr solution by keeping the silk-to-LiBr ratio at 1:4 (i.e., 1 g of silk in 4 mL of LiBr solution). Next, the highly viscous aqueous solution of Bm silk fibroin was dialyzed against distilled water for 48 h using a 12 kDa cellulose membrane (Sigma-Aldrich), with regular water changes during the process at every 8 h. The 12 kDa cellulose filter was used because Bm silk fibroin mainly consisted of light and heavy chains having molecular weights of ∼26 and ∼390 kDa, respectively.^31−33^ Components below 10 kDa are generally degraded proteins^34^ generated during the dissolution process and can be easily eliminated using a 12–14 kDa dialysis membrane.^35^ The purified silk fibroin solution was then centrifuged at 7000 rpm for 5–7 min at 4 °C to remove any undissolved chunks. The concentration of the regenerated protein was determined by a gravimetric method and stored at 4 °C until further use.

### 2.3 Fabrication of the Bm:GNP-Based Aligned Scaffold

The degummed Bm fibers were aligned over a microscopic glass slide (25 mm × 75 mm) manually and secured in place by affixing adhesive tapes at both longitudinal ends (Figure S1). A minimum of four fiber layers were utilized to create a uniformly aligned platform with a thickness of ∼1 mm. Following alignment, the fibers were cross-linked using a 1% (w/v) silk fibroin solution, followed by an overnight air-drying process. Silk fibroin comprises a unique sequence of amino acids containing a sufficient number of chemically active residues, such as lysine, tyrosine, serine, glutamic acid, histidine, and aspartic acid. These residues are commonly utilized for chemical modifications aimed at tailoring the properties of silk.^36,37^ Consequently, it is anticipated that the amino acid sequences present in both silk fibroin fibers and the solution will engage in intermolecular interactions such as hydrogen bonding or hydrophobic/hydrophilic interactions, facilitating the cohesion of aligned fibers (Figure S3). Subsequently, the aligned scaffold underwent treatment with 70% (v/v) ethanol for 1 h to induce β-sheet transition, which ensures the water insolubility of the coating as well as enhances the stability of interfiber connectivity.^38^

Then, the resultant Bm scaffolds were air-dried overnight. GNPs were coated over these stabilized aligned cross-linked Bm scaffolds by a vacuum drying process. Typically, GNPs were dispersed in water at a concentration of 1 mg/mL through an ultrasonication process for 3 h at a power of 300 W and a frequency of 10 kHz using a probe sonicator (Model: PKS - 500F, PCI Analytics, India) as well as to achieve maximum exfoliation of the graphene flakes. The resultant GNP solution was then drop-cast over the cross-linked Bm scaffolds and vacuum-dried at 60 °C for 24–30 h. The drop-casting of the GNP solution was repeated three times (performed at every 3 h interval after the drying process started) to achieve a uniform and continuous coating. The resultant GNP-coated Bm scaffolds, i.e., Bm:GNP, were reduced under constant shaking in a 1 mg/mL l-Aa solution for 24 and 48 h to remove the oxygen-containing functional groups in GNPs. The reduced Bm:GNP scaffolds for 24 and 48 h were designated as Bm:rGNP-24 and Bm:rGNP-48, respectively. A detailed sample designation with corresponding descriptions is provided in Table S1 (Supporting Information).

### 2.4 Physicochemical Characterization

The surface morphology of the fabricated scaffolds was characterized by using a field emission scanning electron microscope (SIGMA VP FESEM, ZEISS). The crystallinity of the scaffolds was tested by an X-ray diffractometer (Rigaku, 007HF, Japan) with Cu Kα radiation (λ = 1.54 Å) at room temperature at an angular range of 10–60° in 2θ, in steps of 0.050°. Chemical compositional analysis was conducted using a Fourier transform infrared (FT-IR) spectrophotometer (Bruker VERTEX 70 FT-IR spectrophotometer, Germany) and a micro-Raman spectrometer (LabRAM HR UV–vis NIR, Horiba) to record the FT-IR and Raman spectra, respectively. Mechanical properties of the scaffolds were assessed using a Universal Testing Machine (Tinius Olsen 5ST) equipped with a 2.5 kN load cell at a crosshead rate of 1 mm/min and a gauge length of 20 mm, following a standard ASTM D638 procedure. Steady-state current–voltage (*I*–*V*) measurements were performed using a Keithley 2450 source meter using a two-probe technique at a DC voltage sweep from −10 and +10 V at room temperature.

### 2.5 Enzymatic Degradation Study

The biodegradability of the different silk-based aligned fibrous scaffolds was assessed in the presence of protease XIV from *Streptomyces griseus* (Sigma-Aldrich, ≥3.5 U/mg) at 37 °C.^39^ Protease XIV refers to a nonmammalian enzyme blend employed for in vitro degradation of silk fibroin, exhibiting activity specifically in breaking down β-sheet crystalline structures.^40^ Hence, it has been documented as the most effective proteolytic enzyme for breaking down silk fibroin across a wide range of material formats, such as fibers, films, sponges, and hydrogels. The scaffolds were initially weighed and subsequently subjected to incubation in PBS with 2 U/mL protease XIV.

At regular 15-day intervals of up to 60 days, the scaffolds were rinsed, allowed to air-dry, and then weighed. Throughout the experimental duration, both PBS and protease solution were replaced every 5 days.

The percentage of remaining mass after incubation was calculated by using the following formula.
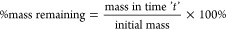


After a 60-day period, the material’s stability was evaluated by examining the altered surface morphology through FESEM and by measuring electrical conductivity via *I*–*V* measurements. The scaffolds underwent two washes to eliminate salts prior to FESEM analysis. *I*–*V* measurements were conducted to ascertain the electrical conductivity under the physiological conditions. This study employed three replicates of each sample.

### 2.6 Cell Culture

Rat Pheochromocytoma PC12 cells (adherent type; *P* = 4) were received from Cell Repository, National Centre for Cell Science, Pune, India, and used for axonal growth potential on the different silk-based scaffolds. The cells were maintained in a growth medium consisting of Ham’s F-12 nutrient mix medium with 10% horse serum, 5% FBS, and 1% penicillin–streptomycin solution at 37 °C in 5% CO_2_. The cells were passaged when they reached 70–80% confluency using a 0.25% trypsin–EDTA solution. For the neural differentiation study, the cells were maintained in the growth medium until 24 h. After that, the growth medium was replaced with the differentiating medium consisting of an F-12 nutrient mix medium supplemented with 1% horse serum, 1% penicillin–streptomycin, and 100 ng/mL NGF-β.

### 2.7 Electrical Stimulation of PC12 Cells

For electrical stimulation (ES) purposes, a homemade setup in a 24-well cell culture plate was used (Figure S2). Briefly, the wells in a 24-well plate (chosen for ES through scaffolds) were linked together using a platinum (Pt) wire of a diameter of 0.5 mm. The wire was affixed horizontally at the bottom surfaces of these wells. Subsequently, the silk-based conductive scaffolds were positioned over the Pt wire within these wells, ensuring direct contact between the bottom surfaces of the scaffolds and the wire. In each well, another Pt wire was placed vertically at a 1 cm distance apart from the scaffolds/horizontal Pt wire. The vertically placed Pt wires were partially dipped in the cell culture medium.

For the neural differentiation study, 2 × 10^4^ cells were seeded on each of the different silk-based scaffolds (size: 10 mm × 10 mm) in a 24-well plate and maintained in the growth medium. After 24 h, the growth medium was replaced with the differentiating medium and was counted as Day 1. Next, a pulsed ES with a frequency of 50 Hz and a pulse width of 1 ms with different amplitudes of 100 and 300 mV/cm was applied to the cells using an arbitrary function generator (AFG1022, Tektronix) for 2 h/day until 3 consecutive days starting from Day 1 to Day 3. Nonstimulated cells grown in the same condition were treated as control. The culture was continued until Day 10 for the axonal growth study. The experiments were repeated three times with *n* = 3.

### 2.8 β(III) Tubulin Immunostaining

To affirm the neuronal differentiation and assess the neurite outgrowth of PC12 cells, electrically stimulated and nonstimulated cells were stained using neuronal markers—anti-β(III) tubulin (Abcam, ab18207) after 10 days. For that, the cell-laden scaffolds were rinsed with 1× PBS three times and fixed with 100% methanol (ice cooled) for 5 min, followed by washing with PBS. The cells were permeabilized using 0.1% (v/v) Triton X-100 (Sigma-Aldrich) in PBS for 15 min. After washing, the cell-seeded scaffolds were incubated with 2% bovine serum albumin (BS, Sigma-Aldrich) in 0.1% Tween PBS (PBST, Sigma-Aldrich) for 1 h. Then, the cell-laden scaffolds were treated with 5 μg/mL of anti-β (III) tubulin (1:1000) in PBST overnight at 4 °C followed by washing with PBST and incubation with 4 μg/mL secondary antibody goat antirabbit Alexa Fluor 488 (ab150077, 1:500) for 1 h at room temperature. Then, the cells were counterstained with 1 μg/mL DAPI (1:2000) in PBST for the nucleus for 5 min. The cell-laden scaffolds were directly visualized under a fluorescence microscope (EVOS M7000 Imaging System, Thermo Fisher), and representative images were presented.

### 2.9 Assessment of the Intracellular Ca^2+^ Level

PC12 cells were seeded onto pure Bm, Bm:GNP, and Bm:rGNP-48 at a density of 1 × 10^4^ cells/well and subjected to neural differentiating medium after 24 h in growth medium. ES was applied to the cells cultured on electroconductive scaffolds as described in Section 2.7. Immediately after ES on Day 1, both nonstimulated and stimulated cell-laden scaffolds were washed with PBS thrice. Then, 5 mM fluo-4-acetoxymethyl (Fluo-4 AM) ester in DMSO was diluted to 5 μM in PBS. The cell-laden scaffolds were incubated at 37°C for 60 min in the diluted Fluo-4 AM. Fluorescence images of calcium signals (stained in green) were studied using a fluorescence microscope (EVOS M7000 Imaging System, Thermo Fisher).

### 2.10 Statistical Analysis

All tests with a minimum of *n* = 3 were replicated. Statistical analysis was performed using GraphPad Prism 10 software. Student’s *t*-test or one-way or two-way analysis of variance (ANOVA) was used where appropriate to evaluate the statistical significance. For multiple comparisons, Turkey’s test was performed along with ANOVA. Statistical significance was defined at *p* < 0.05. Results are presented as mean ± standard deviation (SD).

## 3 Results and Discussion

### 3.1 Scaffold Morphology and Structure

**Figure 1:**
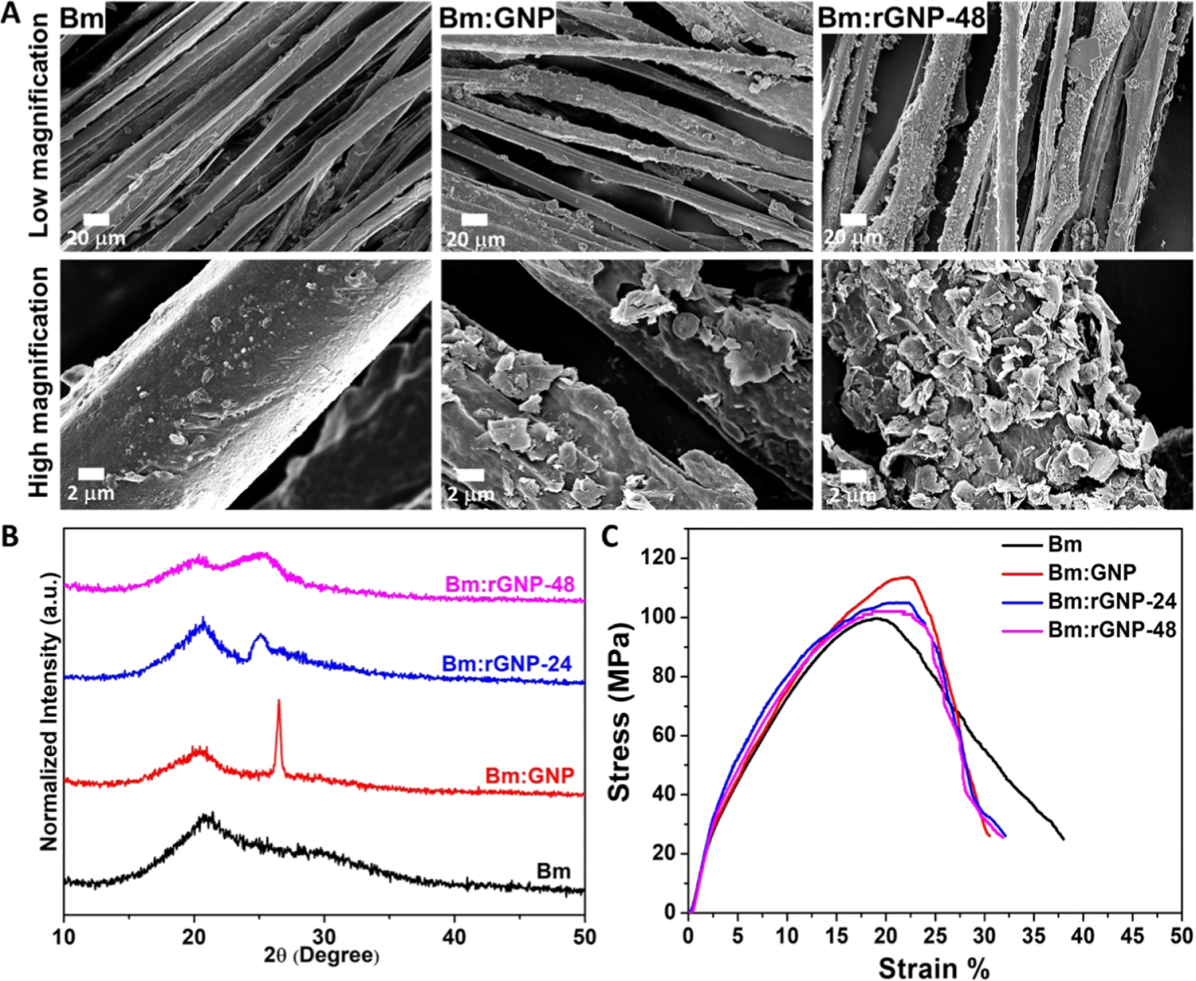
(A) FESEM images of aligned scaffolds of the degummed Bm scaffold, Bm:GNP, and Bm:rGNP-48 as indicated. Upper panel: low-magnification images showing the overall surface morphology of the scaffolds (scale bar = 20 μm) and lower panel: high-magnification images (scale bar = 2 μm) showing the surface morphology of the individual fibers. (B) XRD spectra showing the diffraction patterns of Bm:GNP scaffolds before and after reduction for 24 and 48 h along with the pure Bm scaffold. (C) Mechanical behavior of Bm, Bm:GNP, Bm:rGNP-24, and Bm:rGNP-48 scaffolds shows their stress versus strain response.

The FESEM images demonstrate clear evidence of the well-organized aligned morphology of the various silk-based scaffolds (Figure 1 (A)). The pure Bm scaffold exhibits a relatively smoother surface when compared to the GNP-coated scaffolds. The observed minor surface roughness indicates the presence of a silk fibroin coating over the fibers. The silk fibroin coating induces sufficient stability of the interfiber connectivity, as evident from the FESEM images (Figure S4). The vacuum coating process effectively introduced several layers of the GNP coating, producing electroconductive microfibers having diameters of 17.65 ± 2.91 μm (Bm:GNP) and 18.27 ± 5.75 μm (Bm:rGNP-48), whereas the Bm scaffold possesses a fiber diameter of 13.63 ± 2.01 μm. The visual observation of the increased surface roughness along with the increased average fiber diameter of the various Bm:GNP-based scaffolds indicates successful GNP coating over the Bm scaffolds. This also reveals that the GNPs present on the fibers prior to reduction (i.e., in Bm:GNP) cluster together, in contrast to the more dispersed and exfoliated reduced GNPs present in Bm:rGNP-48. This can be attributed to the capping behavior of l-Aa in addition to its well-known reduction capability, as previously reported.^10^ The oxidized products of l-Aa potentially stabilize reduced GNPs (rGNPs).

X-ray diffraction (XRD) analysis uncovers a broad yet strong diffraction peak at ∼20° present in the diffraction patterns of all scaffolds, corresponding to the β sheet crystalline structure of Bm corresponding to a *d*-spacing of 4.31 Å (Figure 1(B)).^41,42^ The broad pattern around ∼30° in the diffractogram of Bm indicates the presence of a random coil structure of Silk I, corresponding to a *d*-spacing of 3.16 Å.^43^ The GNPs used in this study appear clustered in the form of GNP sheets, which resemble graphite powder. GNP-coated Bm fibers before reduction, i.e., Bm:GNP, display a strong sharp peak at 2θ = 26.5°, corresponding to the diffraction plane of graphite with a *d*-spacing of ∼3.44 Å.^44^ Nonetheless, after reduction using l-Aa for 24 and 48 h, a new broad diffraction peak gradually appears at around 25°, corresponding to a *d*-spacing ∼3.7 Å. This is a signature peak of the reduced form of graphene sheets, as reported earlier.^10,45^ The removal of oxygen-containing functional groups after reduction leads to randomly organized graphene sheets with a higher defect density. As a result, the amorphicity increases, which is evident from the highest peak broadening in the case of Bm:rGNP-48.

### 3.2 Mechanical Behavior

A biomaterial scaffold intended for use as an electroconductive nerve guidance channel (NGC) ideally should be mechanically robust enough to withstand physiological loads as well as stiff enough to support suturing and electrodes for external ES.^46^ Therefore, the aligned scaffolds were subjected to tensile strength tests to assess their mechanical properties. Stress versus strain curves of aligned scaffolds and characteristic parameters derived from these are shown in Figure 1(C) and Table 1, respectively. The degummed Bm fibers obtained from cocoons have previously been shown to possess elastic moduli in the range of 1–10 GPa, depending on their diameters.^47^ The Bm fibrous scaffold reported herein also demonstrates similar mechanical behavior, while GNP-coated Bm scaffolds (after reduction) have a slightly higher elastic modulus (Table 1). Nonetheless, no statistically significant differences in the ultimate tensile strength and % elongation at break are found among various types of scaffolds.

**Table 1:** Mechanical Properties of Pure Bm and Different Variants of Silk:GNP-Based Scaffolds

sample name	elastic modulus (GPa)	ultimate tensile strength (GPa)	elongation at break (%)
Bm	1.23 ± 0.14	0.098 ± 0.001	37.95 ± 0.07
Bm:GNP	1.41 ± 0.44	0.106 ± 0.005	36.47 ± 1.36
Bm:rGNP-24	1.51 ± 0.62	0.102 ± 0.003	31.9 ± 0.35
Bm:rGNP-48	1.47 ± 0.21	0.099 ± 0.003	35.6 ± 0.42

### 3.3 FT-IR and Raman Spectroscopy

**Figure 2:**
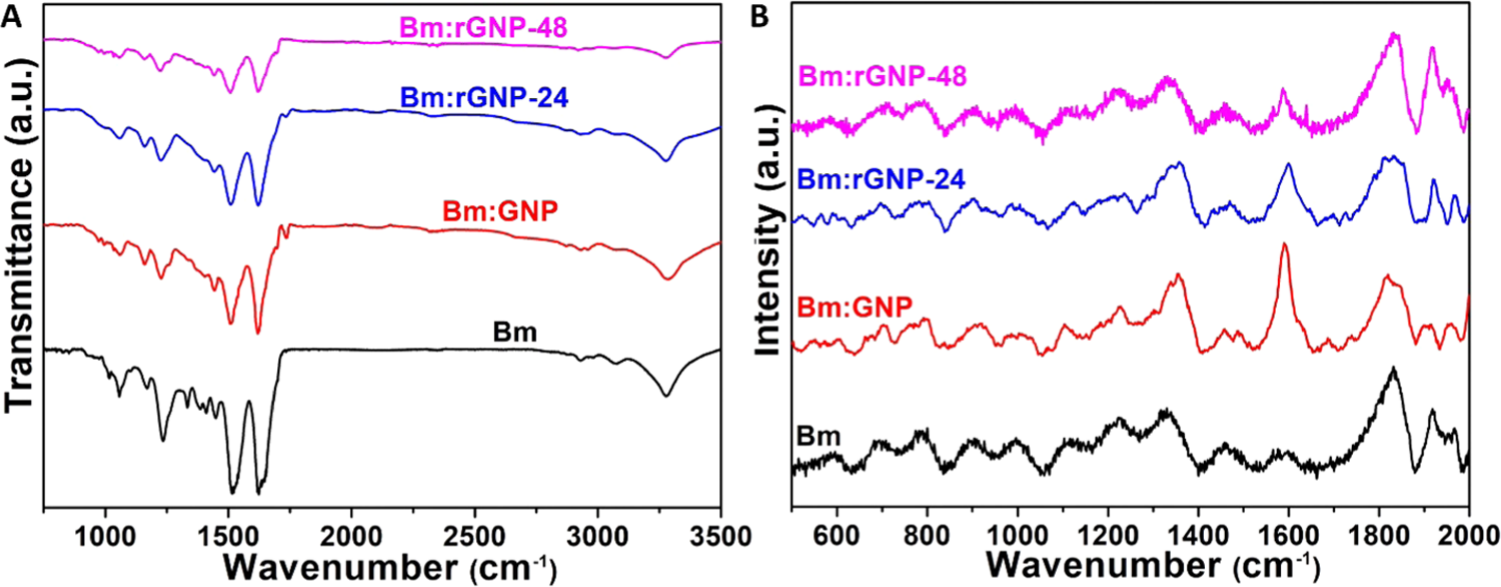
(A) FT-IR patterns, and (B) Raman spectra of pure silk (Bm) and different variants of Bm:GNP-based scaffolds before and after reduction with ascorbic acid (Aa).

ATR FT-IR spectra of pure silk and various aligned silk:GNP-based fibrous scaffolds (before and after reduction) exhibit the dominant presence of characteristic fundamental vibrational bands associated with silk fibroin (Figure 2(A)). The Bm scaffold, which underwent treatment involving 1% regenerated Bm silk fibroin followed by 70% ethanol, exhibits a predominant β-sheet structure, characterized by a prominent peak at 1618 cm^–1^ corresponding to amide I vibration.^48^ Additionally, the presence of α-helix/random coil structures is indicated by a shoulder peak observed at 1650 cm^–1^. The characteristic amide II and III peaks are also present within the ranges 1515–1538 and 1238–1333 cm^–1^, respectively, representing the combined C–N stretching and N–H bending of the secondary structure of silk fibroin. Nevertheless, in various Bm:GNP-based scaffolds, the intensity of these distinctive peaks representing silk secondary structures is diminished, indicating the successful coating of the silk fibers with GNPs. This observation aligns with the findings of surface morphological evaluations (as shown in Figure 1(A)). Reduction of GNP using l-Aa effectively removed oxygen-containing functional groups, as demonstrated in our previous study.^44^ The vibrational bands corresponding to the O–H stretching of hydroxyl (∼3300–3400 cm^–1^), C=O stretching of carboxyl (1736 cm^–1^), C–OH bending (1407 cm^–1^), C–O–C stretching in epoxide (1235–1296 cm^–1^), and C–O stretching of alkoxy (1061–1100 cm^–1^) present in the FT-IR spectrum of GNP gradually disappear or weaken after its reduction for 24 and 48 h (Figure S5). Most of these bands tend to coincide with the characteristic vibrations of silk fibroin, making them less discernible in Bm:GNP, with the exception of the prominent C=O stretching at 1731 cm^–1^. Nonetheless, the complete absence of this peak in Bm:rGNP-48 provides conclusive evidence of the effectiveness of the reduction process. It is worth mentioning that vibrations related to aromatic C=C stretching of the sp^2^-hybridized carbon lattice of GNPs appear around 1630 cm^–1^, which overlaps with the characteristic amide I vibration of silk fibroin. Notably, the intensity of this peak diminishes progressively with the prolonged reduction process employing l-Aa. These findings align closely with previous research on the reduction of GO using l-Aa.^10,44,49^

Raman spectra of different samples as obtained within a wavenumber range of 500–1800 cm^–1^ are presented in Figure 2(B). All of these spectra display the characteristic molecular conformations of Bm silk fibroin with the characteristic Raman bands for sp^2^ and sp^3^ carbons of GNPs emerging in the silk:GNP composite samples. The presence of the amide I band at 1676 cm^–1^ in the pure Bm scaffold confirms again the β-sheet conformation, further validating the FT-IR and XRD findings discussed earlier.^50^ Interestingly, a strong band at around 1335 cm^–1^ corresponds to alanine-based motifs in Bm, further revealing its β-sheet conformation. This is further supported by the appearance of a strong amide III signal at 1227 cm^–1^. The presence of a moderately intense peak in the range of 1560–1570 cm^–1^ is likely associated with aromatic amino acids, such as phenylalanine, tryptophan, tyrosine, etc., present in the silk fibroin protein chain.^51^ The peak at about 1460 cm^–1^ can be assigned to CH_2_/CH_3_ bending in different polypeptide chains of silk fibroin. The presence of the α-helix/random coil conformation, attributed to degummed silk fibers based on the FT-IR findings, is further supported by the peaks observed at 1105–1120 cm^–1^ (*v*C–C) and 900–990 cm^–1^ (*v*C–N), consistent with prior research.^52^ Much like the findings in our FT-IR studies, the majority of these peaks associated with silk fibroin exhibit a reduction in intensity as a result of the GNP coating on the silk fibers. Raman spectroscopic investigation further indicates significant structural changes of GNP coated over the silk fibers during the reduction process. In the Raman spectra of Bm:GNP, the band at 1590 cm^–1^ is associated with the vibration of sp^2^-hybridized carbon atoms and is designated as G-band.^53^ Conversely, the band at 1350 cm^–1^ corresponds to the well-documented D mode or the phonon mode that represents the conversion from a sp^2^-hybridized carbon to an sp^3^-hybridized carbon. The *I*_D_/*I*_G_ ratio holds significant importance in carbon-based materials and is commonly employed to determine the purity of materials being studied.^10^ The *I*_D_/*I*_G_ ratios for Bm:GNP, Bm:rGNP-24, and Bm:rGNP-48 were determined to be 0.87, 1.00, and 1.03, respectively. It is worth noting that the increase in *I*_D_/*I*_G_ ratio with the extension of reduction time indicates the presence of defects and a partially disordered crystal structure in GNP sheets due to the removal of oxygen-containing functional groups. This is consistent with SEM observations, which showed randomly distributed GNP sheets in Bm:rGNP-48 in contrast to the aggregated GNP sheets in Bm:GNP.

Additional insights from FT-IR and Raman spectroscopic analyses also indicate the presence of interfacial electrostatic or hydrogen bonding between Bm silk fibroin and GNPs, as evidenced by the observed shifts in major peaks associated with protein secondary structures, as reported by Magaz et al.^17,54^ For example, the amide III band in the FT-IR spectra of Bm:GNP and Bm:rGNP-24/48 undergoes a red shift from 1233 to 1225 cm^–1^ and, similarly, the N–H bending vibration in the amide II region red-shift from 1515 to 1505 cm^–1^ after GNP coating. Likewise, the Raman-active amide I band of Bm exhibits a shift from 1676 to 1684 cm^–1^ in various Bm:GNP-based scaffolds, both before and after reduction. In the cases of Bm:rGNP-24 and Bm:rGNP-48, the amide III band undergoes a shift to 1220–1240 cm^–1^, accompanied by significant broadening. The CH_2_/CH_3_ bending in polypeptide chains of Bm exhibits two maxima in the region 1430–1460 and 1480 cm^–1^ in different composite scaffolds.

### 3.4 Electrical Conductivity Studies

**Figure 3:**
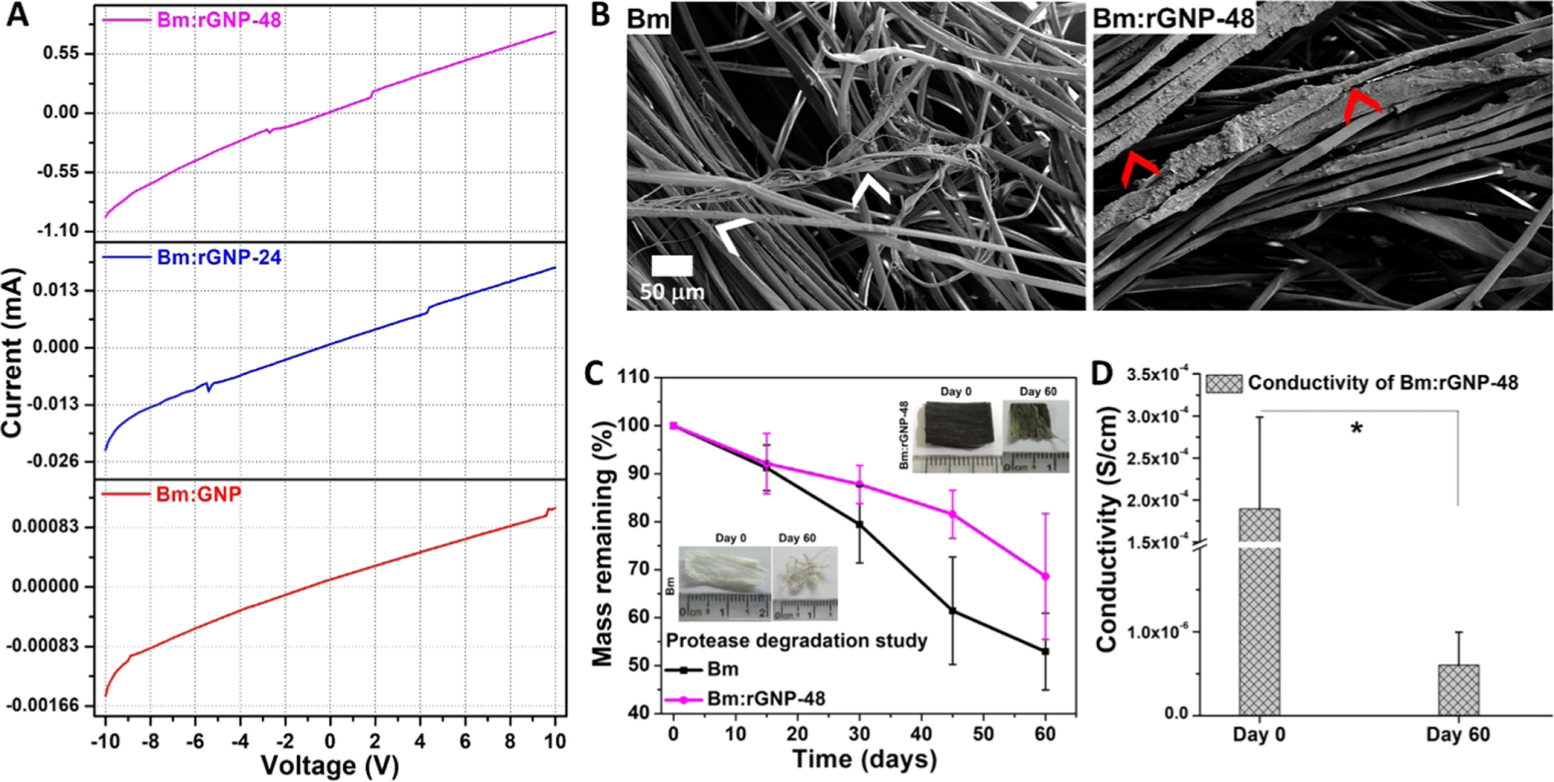
(A). Room-temperature (300 K) *I–V* characteristics of various Bm:GNP-based scaffolds before and after reduction under a potential range from −10 to +10 V. In vitro biodegradation results showing SEM images (B) and residual mass profile (C) of pure Bm and Bm:rGNP-48 scaffolds incubated in a 2 U/mL protease solution for 60 days. Insets of (C) show photographs of scaffolds before and after protease treatment as labeled. In (B), white and red arrows indicate the fragmented fibers in the Bm scaffold and partial delamination of rGNPs from the silk fibers under proteolytic action (D). Decreased electrical conductivity of the protease-treated Bm:rGNP-48 in comparison to its untreated counterpart. * indicates statistical significance at *p* < 0.05.

Current–voltage (*I*–*V*) characteristics of different variants of Bm:GNP-based scaffolds demonstrate an increased current value with increasing potential and nearly symmetric behavior under both −ve and +ve bias (Figure 3(A)). Reduced variants of scaffolds, i.e., Bm:rGNP-24 and Bm:rGNP-48, show an increasing trend in the voltage-dependent current with increased reduction time, which is also 10–100-fold higher than the nonreduced Bm:GNP. As a matter of fact, the scaffold subjected to l-Aa reduction for 48 h has about 10^3^ and 10^2^ times greater surface electrical conductivity than Bm:GNP and Bm:rGNP-24, respectively (Table 2). This suggests that the longer reduction time using l-Aa improves the electrical conduction in GNPs through defect creation (supported by XRD) and is consistent with earlier reports.^10,11^ Notably, GNP-coated silk fibers after reduction for 48 h demonstrated a greater electrical conductivity of up to 3 × 10^–4^ S/cm than the conductivity value (∼4 × 10^–5^ S/cm) of blended rGO/silk, reported elsewhere.^17^

**Table 2:** Surface Electrical Conductivity of Different Bm:GNP-Based Scaffolds

sample name	conductivity (S/cm)
Bm:GNP	3.05 × 10^–7^ ± 2.87
Bm:rGNP-24	5.19 × 10^–6^ ± 1.15
Bm:rGNP-48	1.89 × 10^–4^ ± 1.09
Bm:rGNP-48 (protease)	6.05 × 10^–8^ ± 3.90

### 3.5 In Vitro Biodegradation Study

Protease aids in nerve regeneration by clearing out the damaged tissue.^55^ Silk fibroin is highly susceptible to such proteolytic action, which can result in its fragmentation into smaller polypeptides, eventually breaking down into amino acids that can be readily absorbed or metabolized within the body.^40^ Hence, an in vitro biodegradation study of pristine Bm and Bm:rGNP-48 was carried out in protease solution for 60 days, and the results are presented in Figure 3(B–D). The effect of this is evident from the mass degradation profile of the Bm scaffold, which undergoes ∼50% mass loss after 60 days (Figure 3(C)). However, the degradation behavior could be controlled when the silk fibers are coated with GNPs, resulting in only ∼30% mass loss of Bm-rGNP-48. The findings suggest that the GNP coating delays the degradation kinetics of silk fibroin. This observation is substantiated by the FESEM images of the degraded scaffolds, which clearly reveal fiber fragmentation due to proteolytic activity (most prominent in Bm) and the partial removal of the rGNPs coating (Bm:rGNP-48) with relatively intact silk fibers (Figure 3(B)). The partial loss of the rGNP coating leads to a decrease in fiber diameter from 18.27 ± 5.75 μm to 15.04 ± 3.31 μm. As a result, Bm:rGNP-48 showed a decrease in electrical conductivity (by a factor of 10^–4^) when subjected to proteolytic degradation for 60 days. However, the electrical conductivity still falls within the range characteristic of ideal semiconductors or the scaffolds possess better conductivity than bioelectronic conductors like melanin (Figure 3(D) and Table 2).^56,57^ The findings indicate that the GNP coating plays a crucial role in preserving the electrical and structural stability of the scaffolds in the presence of proteolytic activity. This underscores the potential for adjusting the biodegradability of these conductive scaffolds. Such a property holds pertinence in the context of nerve regeneration, which typically demands long-term stability of biomaterials owing to their slower growth rate compared to other tissues. The release of GNPs from Bm:GNP and rGNPs from Bm:rGNP-24 and Bm:rGNP-48 into the aqueous environment was assessed by incubating the different scaffolds in PBS for 16 days at 37°C. The micrographs reveal a minor depletion of the GNP/rGNP coating from the silk fibers (Figure S7(A)), which was also verified by recording the absorbance spectra of PBS (in which scaffolds were incubated) on Days 1 and 16 (Figure S7(B)). The results indicate a slightly higher release of rGNPs as compared to GNPs due to weaker adhesion after reduction, which is in agreement with the previous study.^58^ In biological conditions, the graphene materials were shown to adsorb serum proteins and bind integrin protein on the cell surface.^59,60^ Furthermore, a sustainable approach was utilized to reduce GNPs in this study, ensuring that any slight delamination of graphene materials would not adversely affect the biological performance of the scaffolds, as elaborated on in the preceding sections.

### 3.6 Effect of Intrinsic Electrical Conductivity and Electrical Stimulation on Axonal Growth

There is a consensus that a material’s intrinsic electrical conductivity has a dominating role in electrically stimulated neuronal growth.^61^ Studies have reported that the increased electrical conductivity of l-Aa-reduced electrospun rGO/silk scaffolds contributed to enhanced neurite growth,^17,27^ gap junction formation in cardiomyocytes,^25^ and restoration of electrical coupling in a myocardial infarction rat model.^26^

**Figure 4:**
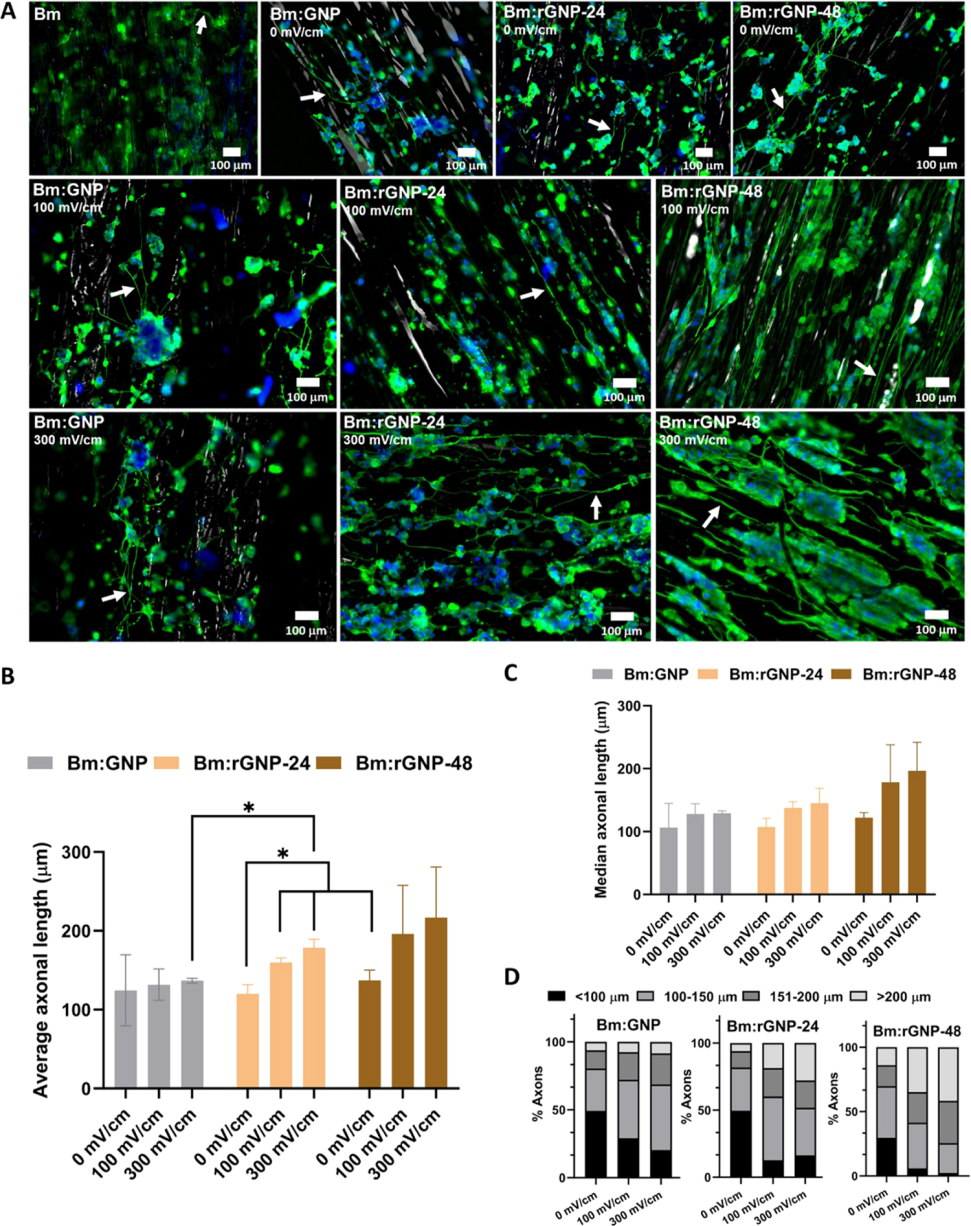
Immunostaining of PC12 cells after 10 days of culture to confirm their neuronal differentiation by the β(III) tubulin neuronal marker, counterstained by DAPI (nucleus staining), which were subjected to pulsed ES with a frequency of 50 Hz and a pulse width of 1 ms with amplitudes of 100 and 300 mV/cm for 2 h/day until 3 consecutive days starting from Day 1 to Day 3. (A). Representative fluorescence images of the differentiated PC12 cells with axonal projections (indicated with white arrows) on different scaffolds as labeled. Scale bar: 100 μm. Quantitative assessment illustrating (B) average axonal length, (C) median axonal length, and (D) axonal length distribution in four specific ranges: <100, 100–150, 151–200, and >200 μm. * indicates statistical significance at *p* < 0.05.

To investigate the impact of varied electrical conductivity (with varied l-Aa-induced reduction times) on neurite growth, the neuronal characteristics of neuronal-like PC12 cells were evaluated on the nonreduced and various reduced silk:GNP-based scaffold under pulsed ES of 100 and 300 mV/cm. Cells were stained with the β(III) tubulin neuronal marker after 10 days of culture to visualize the expression of neuronal characteristics such as growth cone formation, axonal projection, and branching, and representative fluorescence images are shown in Figure 4(A). The results show axonal projection or elongation following the aligned orientation of the fibers in all types of scaffolds including pure Bm. It can be visually noticed that the electrically stimulated PC12 cells seeded on different electroconductive scaffolds possess relatively longer axonal elongation or neurite outgrowth as compared to the nonstimulated cells. To get a clearer picture, the β(III) tubulin-stained images were analyzed by using ImageJ software for the quantitative assessment of the axonal growth.

For this, the axonal length was measured as a linear distance between the cell junction and the tip of a neurite. Axonal length data, which were at least twice the diameter of the cell body, were considered for analysis, and at least N = 100 axons/neurites were analyzed for each sample. Axonal growth data are presented in terms of average axonal length and median axonal length in Figure 4(B,C), respectively. Cells that undergo ES exhibit notably longer axons compared to cells that did not receive stimulation (Figure 4(B,C)). Particularly, Bm:rGNP-48 demonstrates the longest neurite projection compared to Bm:rGNP-24 and Bm:GNP, under both ES and no ES conditions. It is noteworthy that both the average and median axonal lengths are longer under ES on scaffolds with higher electrical conductivity, aligning with the consistent trend observed as Bm:GNP < Bm:rGNP-24 < Bm:rGNP-48. Results further show a greater average axonal length on Bm:rGNP-48 than that on Bm:rGNP-24 under no ES, which is statistically different at *p* < 0.05 (two-way ANOVA). Similarly, statistical significance also exists between Bm:rGNP-24 and Bm:GNP under ES at an amplitude of 300 mV/cm. Nonetheless, there is no statistical significance in axonal growth on Bm:rGNP-48 under ES with others due to large extremities in axonal growth distribution. It can be associated with the higher density of cells forming a comparatively higher number of neurites on Bm:rGNP-48 under ES, which can also be seen from the β (III) tubulin-stained images. This is also supported by the observation that the number of neurites available for analysis on Bm:rGNP-48 subjected to ES ranges from 150 to 180, while this value is <140 on the other ES-treated groups under the same field of view. β(III) tubulin-stained images further depict that there is a tendency among the cells to aggregate or to form clusters across the scaffold when electrically stimulated, and this tendency is more pronounced in the case of Bm:rGNP-48 (both at 100 and 300 mV/cm) and to some extent on Bm:rGNP-24 (at 300 mV/cm). This can be correlated with the electrophoretic accumulation of serum proteins of the media on the scaffolds, induced by the externally applied electrical stimulus, as demonstrated by previous studies.^62,63^

A more robust picture of the axonal growth distribution can be obtained by the median axonal length values. Unlike the average axonal length (which is affected by extreme values), it gives insights into the central tendency of the data distribution. Consistent with the previous trend, Bm:rGNP-48 overall has a longer axonal length distribution, indicating their accelerated growth, when compared to all other groups under both ES and no ES (Figure 4(C)).

To further substantiate this finding, the distributions of axonal lengths were assessed within four specific ranges: <100, 100–150, 151–200, and >200 μm (Figure 4(D)). This analysis reveals a notable increase in the number of axons with longer projections across all groups subjected to ES. Particularly, scaffolds with higher electrical conductivity have a greater proportion of axons with extended projections, consistently following the pattern observed as Bm:GNP < Bm:rGNP-24 < Bm:rGNP-48, both in the presence and absence of ES. Notably, Bm:rGNP-48 under ES displays a distinct difference compared to all other groups, highlighting the combined effect of enhanced conductive properties and the externally applied electrical stimulus. Quantitative analysis revealed that under ES at 100 and 300 mV/cm, Bm:rGNP-48 comprises 35 and 41% of axons with lengths exceeding 200 μm, respectively. In contrast, the ES-treated Bm:rGNP-24 and Bm:GNP promote only 18–28 and 7–9% of axons with lengths over 200 μm, respectively.

**Figure 5:**
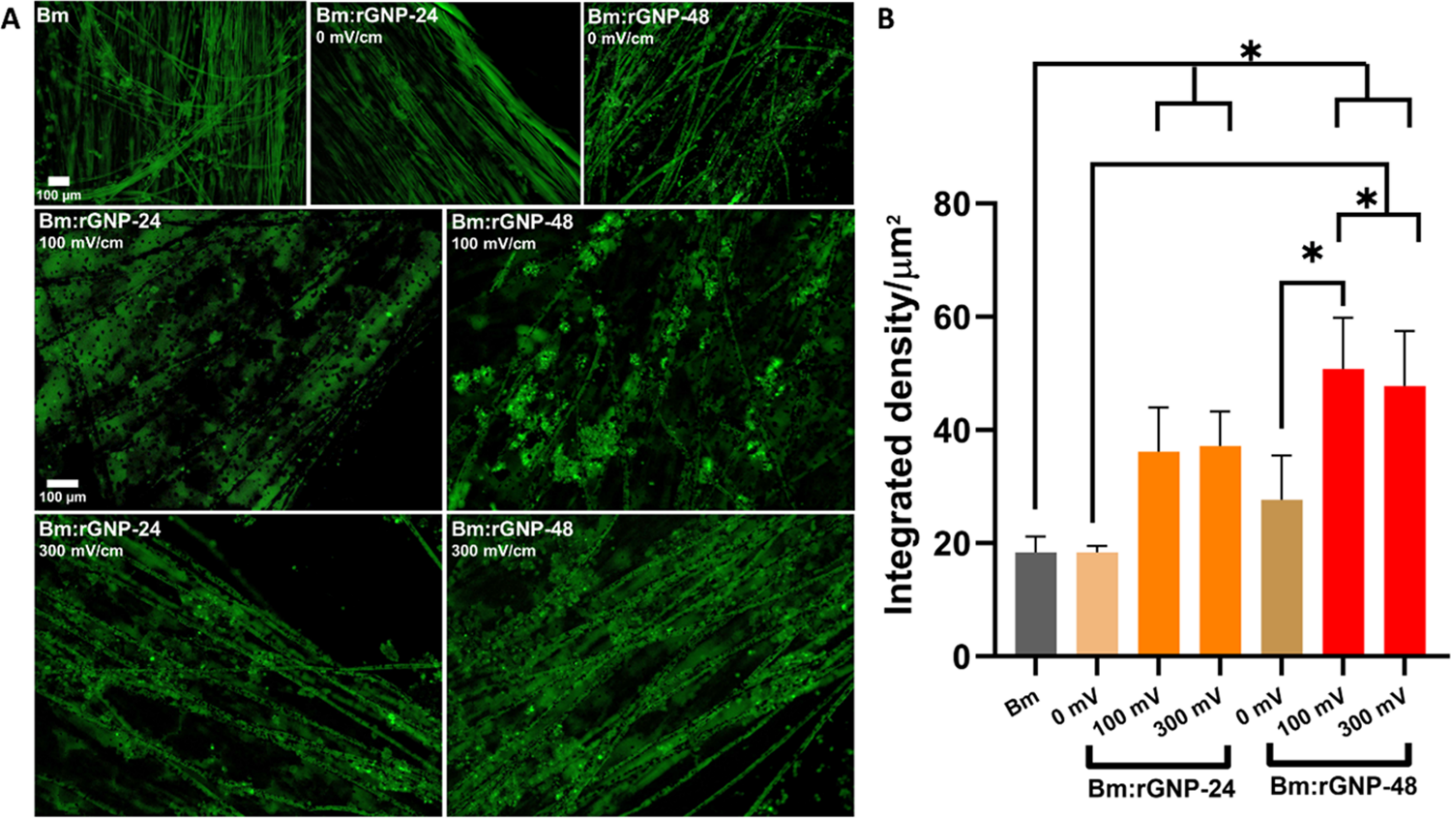
Intracellular Ca^2+^ signaling study after 2 h of ES on Day 1. (A). Representative fluorescence images of PC12 cells stained with Fluo-4 AM seeded on pure silk (Bm) and reduced variants of Bm:GNP scaffolds. Scale bar: 100 μm. (B). Fluorescence (green) intensity expressed in terms of integrated density (using ImageJ software) to quantify the intracellular Ca^2+^ level. * indicates statistical significance at *p* < 0.05.

It has been well established that ES-mediated Ca^2+^ influx plays a crucial role in f-actin polymerization at the growth cone of a regenerating axon.^64^ Externally applied ES can change the steady-state transmembrane potential of neurons and evoke action potential, which affects the ion influx through the membrane to condition the intracellular signal transduction pathways through second messengers such as Ca^2+^, which in turn regulate enzyme phosphorylation and gene expression.^65^ Hence, intracellular Ca^2+^ dynamics is an important indicator to understand the regulating mechanisms of electrically stimulated axonal growth. To accomplish this, PC12 cells seeded on scaffolds prepared using the l-Aa reduction protocol specifically, Bm:rGNP-24 and Bm:rGNP-48, were treated with the Fluo-4 AM dye immediately following ES on Day 1. The resulting green fluorescence, signifying Ca^2+^ expression, was captured with varying intensities (Figure 5(A)). The fluorescence images were analyzed using ImageJ software to measure the integrated density manually, as shown in Figure 5(B). Fluorescence intensity profiles demonstrate a stronger Ca^2+^ expression in PC12 cells subjected to ES when compared to that of the nonstimulated cells. In terms of the scaffold variants, cells seeded on Bm:rGNP-48 have enhanced Ca^2+^ signaling compared to that on Bm:rGNP-24 under both ES and no ES. However, the Ca^2+^ expression is not statistically significant between the different ES-treated groups of both types of scaffolds. In addition, Bm:rGNP-48 shows stronger Ca^2+^ expression under both ES (*p* < 0.0001) and no ES when compared to that on the pure Bm scaffold (Figure 5(A,B)). Ca^2+^ signaling is also higher on ES-treated Bm:rGNP-24 when compared to that on the Bm scaffold, and the results are statistically significant at *p* < 0.05. As mentioned above, enhanced Ca^2+^ signaling in electrically stimulated neuronal-like PC12 cells on Bm:rGNP-48 is believed to contribute to accelerated f-actin polymerization or microtubule formation, leading to superior axonal growth,^66^ as observed in the axonal growth analysis of β(III) tubulin immunostaining results (Figure 4(A–D)).

These observations verify our hypothesis that the difference in the electrical conductivity of the scaffolds has a major role in axonal growth, and in the present case, it is controlled by the reduction protocol employed, more specifically (discussed in Section 3.4) the reduction period. For instance, Bm:GNP, which was not subjected to the reduction process, exhibited the lowest electrical conductivity of the order of 10^–7^ S/cm and demonstrated the lowest axonal growth when compared to that on Bm:rGNP-24 and Bm:rGNP-48, having higher electrical conductivity. Thus, it is evident that scaffolds with better electrical conductivity contribute to enhanced and accelerated axonal growth, which is more prominent under the effect of the external ES. The output pulsed voltage signals applied through the function generator during ES at amplitudes of 100 and 300 mV (at a frequency of 50 Hz and a pulse width of 1 ms) were recorded using an oscilloscope [TBS1072B-EDU, Tektronix], and representative signals are presented in Figure S8. Output peak voltage(s) delivered through Bm:GNP is more distorted (more at 100 mV) and lower as compared with those through Bm:rGNP-24 and Bm:rGNP-48, which can be assigned to their highly resistive nature. In order to confirm that the current values at bias voltages of 100 and 300 mV across these scaffolds in the ES setup were recorded using a source meter under the same condition of ES, i.e., when the scaffolds are in direct contact with neural differentiating media and a Pt wire (Figure S9). Bm:GNP exhibits the lowest current (14.54 ± 6.4 and 17.74 ± 7.04 μA at 100 and 300 mV, respectively) compared to Bm:rGNP-24 (16.67 ± 3.95 and 22.66 ± 6.33 μA at 100 and 300 mV, respectively) and Bm:rGNP-48 (19.39 ± 5.76 and 33.57 ± 4.3 μA at 100 and 300 mV, respectively). This suggests that at identical ES input parameters, the strength of the stimulus delivered to the cells through the different scaffolds differs depending on their intrinsic resistive/conductive behavior. In this context, Bm:rGNP-48 (subjected to the l-Aa reduction protocol for a longer time as compared to the others) has higher current and voltage signal delivery during ES, leading to enhanced and accelerated axonal growth. Furthermore, across all scaffold variations during ES at various potentials or amplitudes, there is successful delivery of the electrical stimulus capable of inducing a current flow exceeding 10 μA, a level shown to be adequate for initiating neural differentiation in PC12 cells or accelerating axonal growth.^67^ Hence, all of these scaffolds displayed enhanced neuronal characteristics under ES compared to those under no ES, but with a distinction between these in terms of the axonal growth efficiency, which is closely associated with their intrinsic conductive properties.

## 4 Conclusions

The present study demonstrates the fabrication of electrically conductive aligned fibrous scaffolds using degummed Bm silk fibers, uniformly coated with GNPs, followed by optimization of electroconductivity through reduction of the coated GNPs using an environmentally friendly, biologically compatible reducing agent (l-Aa). A simple vacuum coating strategy was employed to achieve a uniform coating of GNPs over the Bm scaffold, as evidenced by a surface morphological study using FESEM. The superior coating efficiency is due to the interfacial electrostatic or hydrogen bonding interactions between the Bm silk fibroin and GNPs, as suggested by the FT-IR and Raman spectroscopic analyses. A systematic investigation of the effect of varied reduction periods on the physicochemical properties of the scaffolds was performed, specifically on the electrical conductivity, and the functional impact of this altered property was studied in the in vitro electrically stimulated axonal growth of PC12 cells. A reduction period of 48 h was shown to elevate the surface electrical conductivity of the scaffold by 100–1000-fold when compared to those that are reduced for 24 h (i.e., Bm:rGNP-24) or not subjected to any reduction process (i.e., Bm:GNP). The Bm:GNP scaffold subjected to 48 h of the reduction process, termed as Bm:rGNP-48, showed superior surface electrical conductivity of up to 3 × 10^–4^ S/cm to that (∼4 × 10^–5^ S/cm) of the blended rGO/silk porous scaffold, reported earlier.^17^ Furthermore, the reduction process using l-Aa is anticipated to stabilize the reduced GNPs, i.e., rGNPs over the Bm fibers, and thereby addresses one of the potential concerns associated with delamination of graphene-based materials postreduction. This is supported by the reduced mass loss of Bm:rGNP-48 as compared to the pure Bm scaffold during an in vitro biodegradation study under a proteolytic environment for 60 days as well as retained conductivity, which still falls in the range of ideal semiconductors or possesses better conductivity than bioelectronic conductors like melanin. An electrically stimulated axonal growth study using a neuronal-like PC12 cell line demonstrates enhanced and accelerated axonal regeneration on the scaffolds with higher electroconductivity, More specifically, Bm:rGNP-48 displays elevated expression of neuron-specific β(III) tubulin than all other scaffold types as confirmed by the immunostaining results, which can be correlated with the upregulated intracellular Ca^2+^ dynamics. Overall, this research suggests the potential of the reported strategy to achieve a highly conductive, mechanically robust, and stable silk:graphene-based fibrous scaffold using an eco-friendly and biocompatible reducing agent, namely, l-Aa, which might have implications in the functional regeneration of electrically excitable tissues, including nerves, cardiac, and muscles.

## Supporting Information Available

Schematic illustration of the silk:GNP-based scaffold fabrication process with their representative photographs; sample designation with description; schematics of the electrical stimulation (ES) setup; representative photographs of Bm fibers before and after silk fibroin coating; low-magnification FESEM images of various scaffolds showing fiber interconnectivity; FT-IR of GNPs before and after reduction using l-Aa; in vitro biodegradation results of Bm:GNP and Bm:rGNP-24; GNP/rGNP release assessment study in PBS; representative pulsed voltage signals recorded during ES; Current measurements through different scaffold variants in the ES setup (PDF)
