# Supplementary material for: Impact of the Reduction Time-Dependent Electrical Conductivity of Graphene Nanoplatelet-Coated Aligned Bombyx mori Silk Scaffolds on Electrically Stimulated Axonal Growth
Source: ACS Appl Bio Mater. 2024 Mar 19;7(4):2389–401. doi: 10.1021/acsabm.4c00052 (PMC11022174; doi:10.1021/acsabm.4c00052)
Supplement: Supplementary file 1 — mt4c00052_si_001.pdf [file mt4c00052_si_001.pdf]

Supporting Information

The impact of reduction time dependent electrical conductivity of graphene nanoplatelet coated aligned Bombyx mori silk scaffolds on electrically stimulated axonal growth

Jitu Mani Das[1], Jnanendra Upadhyay[2], Michael G. Monaghan[3,4,5,6,¥], Rajiv Borah[1,3,4,5,*]

AUTHOR ADDRESS:

[1]Life Sciences Division, Institute of Advanced Study in Science & Technology, Guwahati, 781035, India

[2]Department of Physics, Dakshin Kamrup College, Kamrup, Assam, 781125, India

[3]Department of Mechanical, Manufacturing and Biomedical Engineering, Trinity College Dublin, Dublin 2, Ireland

[4]Advanced Materials and BioEngineering Research (AMBER), Centre at Trinity College Dublin and the Royal College of Surgeons in Ireland, Dublin 2, Ireland

[5]Trinity Centre for Biomedical Engineering, Trinity College Dublin, Dublin 2, Ireland

[6]CÚRAM, Centre for Research in Medical Devices, National University of Ireland, H91 W2TY Galway, Ireland

*Rajiv Borah, Ph.D. (Corresponding author)

Department of Mechanical, Manufacturing and Biomedical Engineering, Trinity College Dublin, Dublin 2, Ireland

E-mail: borahr@tcd.ie

¥Michael G. Monaghan, Ph.D. (Co-Corresponding author)

Department of Mechanical, Manufacturing and Biomedical Engineering, Trinity College Dublin, Dublin 2, Ireland

E-mail: monaghmi@tcd.ie

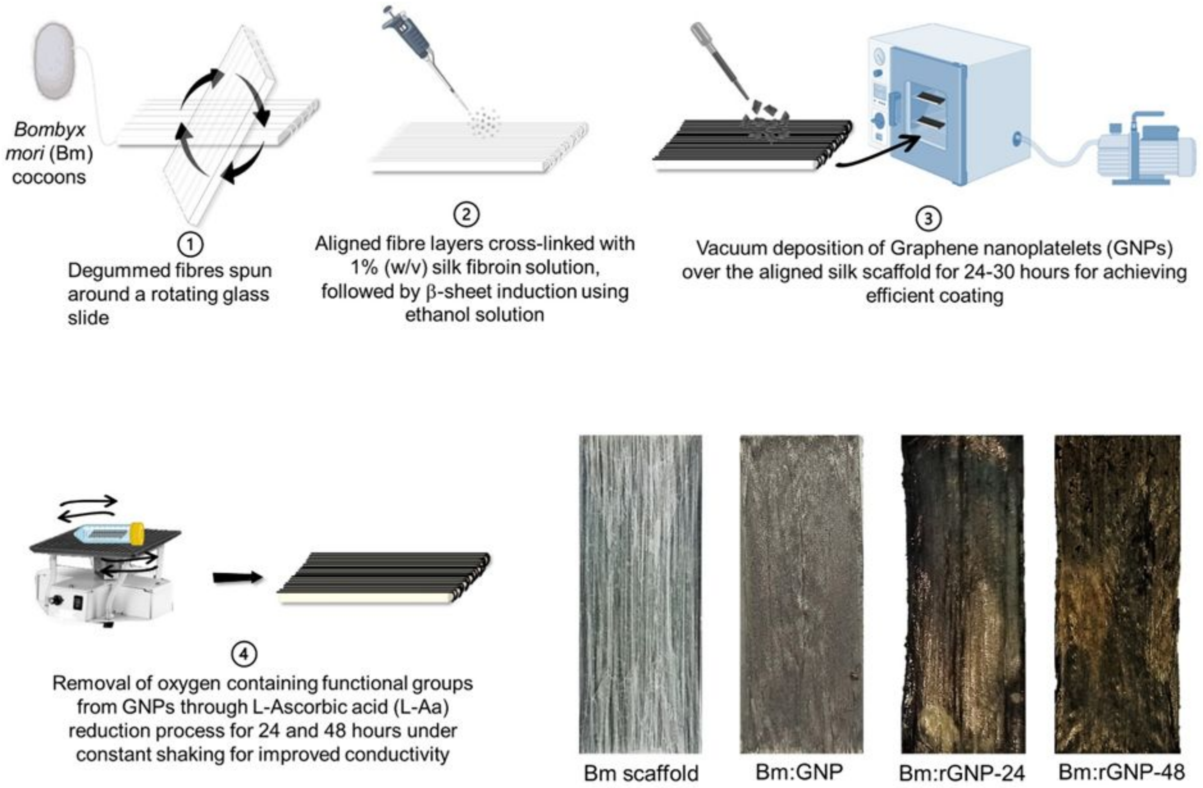

Figure S1: Schematic illustration of fabrication process in sequence (1-4) of aligned microfibrous *Bombyx mori* (Bm) silk scaffolds coated with graphene nanoplatelets (GNPs) followed by eco-friendly reduction using L-Ascorbic acid (L-Aa). Representative photographs of various silk based scaffolds fabricated using the reported process as labelled.

Table S1: Sample designation with description.

Sample designation	Sample description
Bm scaffold	Degummed Bombyx mori (Bm) fibers undergoing cross-linking with 1% (w/v) silk fibroin+70% Ethanol treatment
Bm:GNP	Graphene nanoplatelets (GNPs) coated Bm scaffold (Degummed Bombyx mori (Bm) fibers subjected to cross-linking process using 1% (w/v) silk fibroin+70% Ethanol treatment)
Bm:rGNP-24	Bm:GNP reduced using L-Ascorbic acid (L-Aa) for 24 h
Bm:rGNP-48	Bm:GNP reduced using L-Ascorbic acid (L-Aa) for 48 h

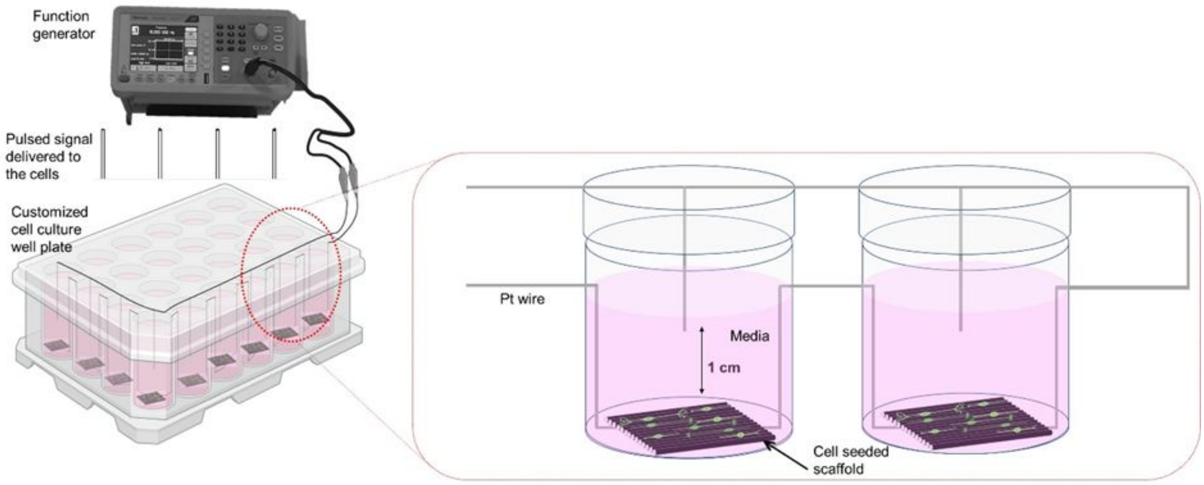

Figure S2: Schematics of electrical stimulation set up showing its different components (e.g. scaffold, media, cell, Pt wire, function generator). Two wells are highlighted in the red rectangle to show the Pt wire connection set-up.

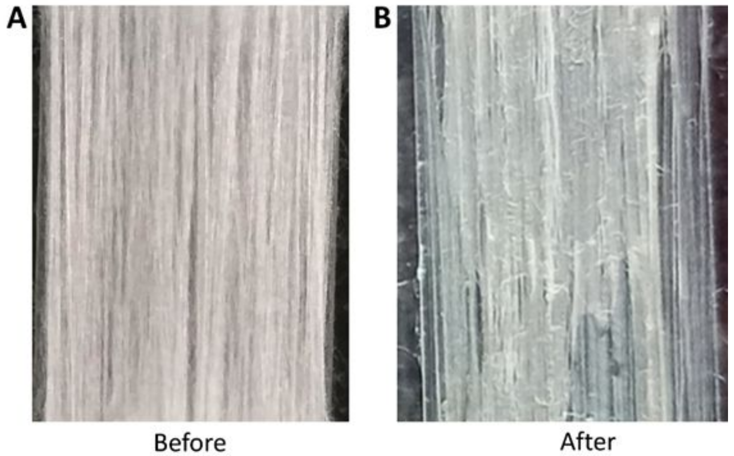

Figure S3: Aligned degummed Bm silk fibers before (A) and after (B) treatment with 1% (w/v) silk fibroin solution followed by 70% ethanol treatment to make the silk-fibroin coating water insoluble through β-sheet induction. After undergoing treatment, the Bm fibers appear to be physically interconnected [in (B)].

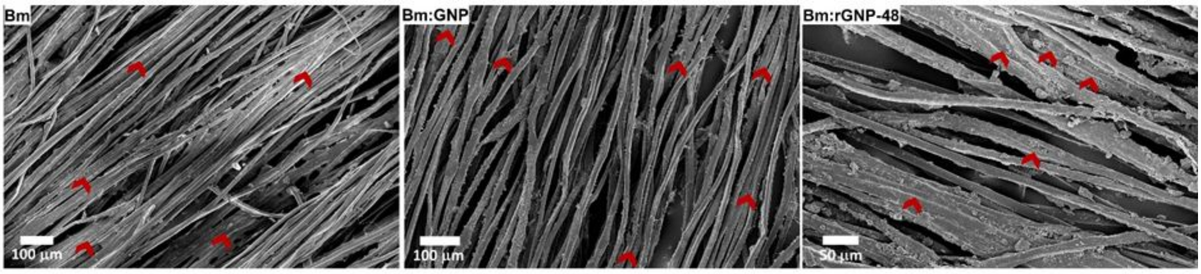

Figure S4: Low magnification FESEM images of aligned scaffolds of degummed Bm scaffold, Bm:GNP and Bm:rGNP-48 as labelled. Red arrows indicate the fiber-interconnectivity.

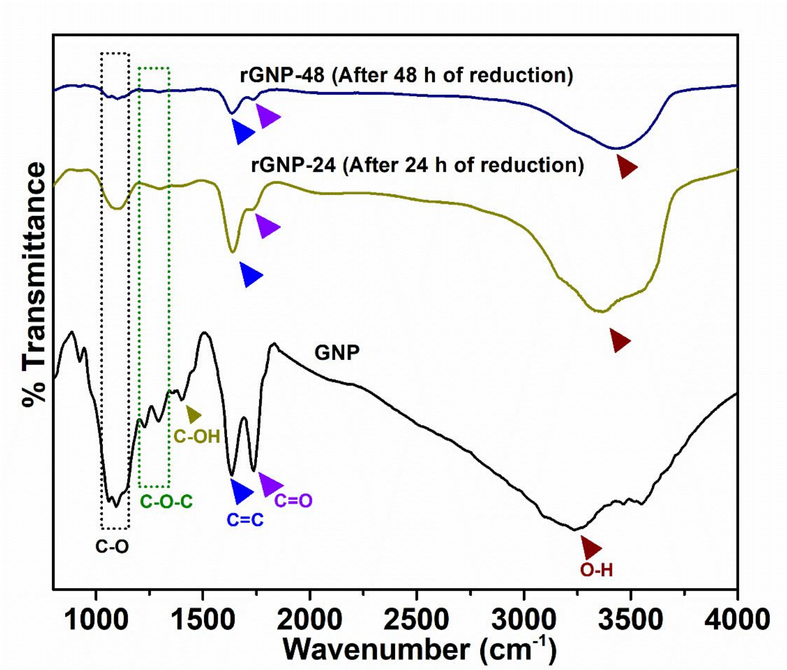

Figure S5: FT-IR of pristine graphene nanoplatelets (GNPs) before and after subjected to 24 and 48 hours of reduction using L-Ascorbic acid (L-Aa).

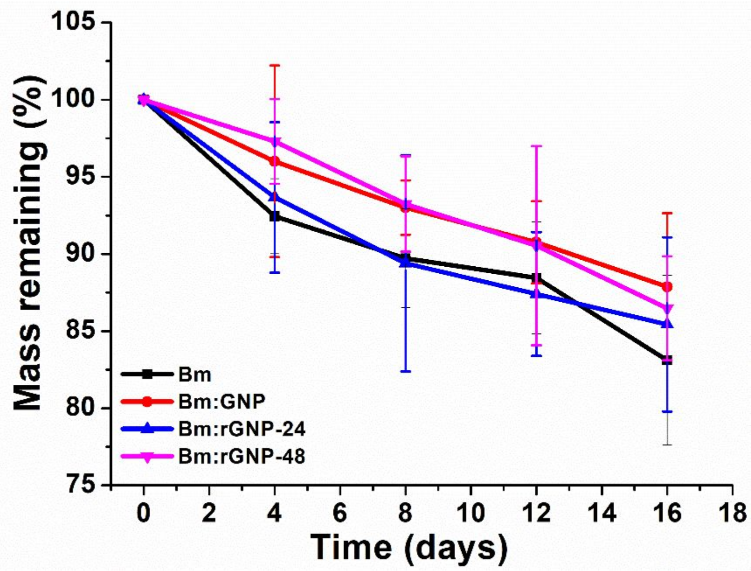

Figure S6: *In-vitro* biodegradation results showing the residual mass profile of Bm, Bm:GNP, Bm:rGNP-24 and Bm:rGNP-48 scaffolds incubated in 2 U/mL protease solution for 16 days. The findings suggest no significant change in enzymatic degradation behavior among the various Silk:GNP based scaffolds.

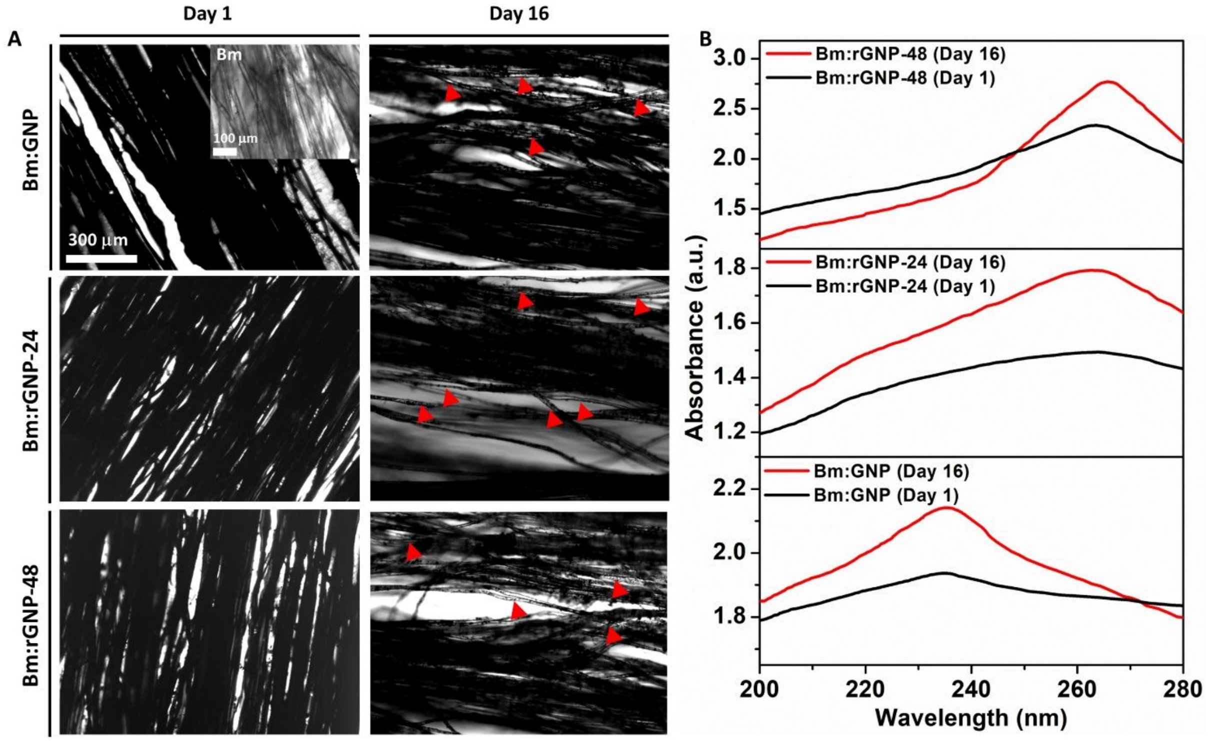

Figure S7: GNP/rGNP release assessment in phosphate buffer saline (PBS) at pH=7.4 and 37˚C. **A.** Representative bright field micrographs of Bm:GNP, Bm:rGNP-24, and Bm:rGNP-48 scaffolds, at Day 1 and Day 16, incubated in PBS. Inset of Bm:GNP shows pure Bm scaffolds, which has more transparency as compared to GNP/rGNP coated scaffolds (appear as black). After Day 16, the minor delamination of GNP/rGNPs from the silk fibers is indicated using red arrows. The delaminated GNP/rGNPs were detected using UV-Vis absorption spectra as shown in **(B)**. GNPs (i.e., without subjected to reduction process) show absorbance centered around 230 nm, whereas rGNPs (after reduction using L-Aa) exhibit absorbance centered around 260 nm [1, 2]. The difference in absorbance intensity of GNPs (released from Bm:GNP) on Day 1 and Day 16 is relatively less as compared to those of rGNPs (released from Bm:rGNP-24 and Bm:rGNP-48). It indicates slightly lesser delamination of GNPs due to stronger adhesion/interaction with the silk fibers (due to presence of oxygen containing functional groups before reduction).

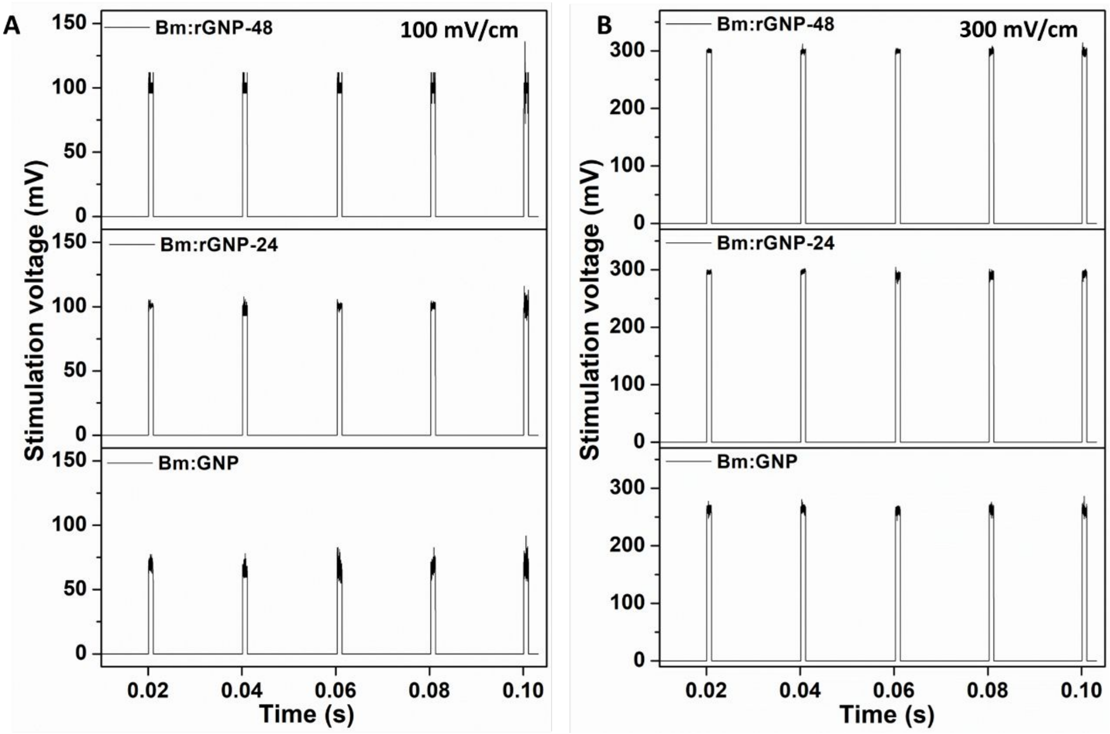

Figure S8. Representative pulsed voltage signals at amplitude of (A) 100 mV and (B) 300 mV, recorded during ES through Bm:GNP, Bm:rGNP-24 and Bm:rGNP-48 scaffolds.

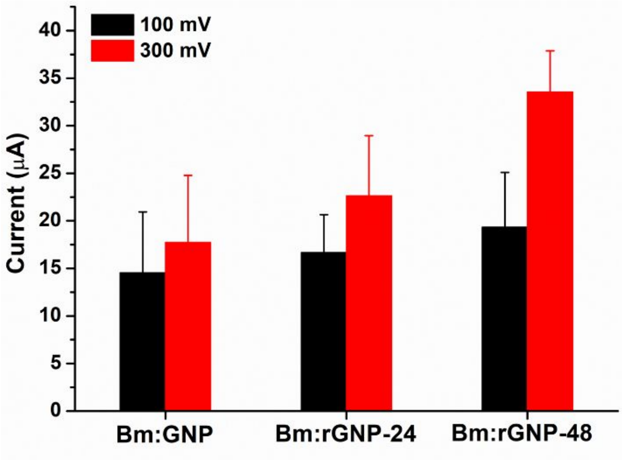

Figure S9: Current values measured at bias voltage 100 and 300 mV across the different scaffolds' variants in the ES set up (scaffolds are in direct contact with neural differentiating media and Pt wire), recorded using a source meter.

References

(1) Alawi, O. A.; Kamar, H. M.; Mallah, A. R.; Mohammed, H. A.; Sabrudin, M. A. S.; Hussein, O. A.; Kazi, S. N.; Najafi, G. Graphene nanoplatelets suspended in different basefluids based solar collector: An experimental and analytical study. *Processes* **2021**, *9* (2), 302.

(2) Venugopal, G.; Krishnamoorthy, K.; Mohan, R.; Kim, S.-J. An investigation of the electrical transport properties of graphene-oxide thin films. *Materials Chemistry and Physics* **2012**, *132* (1), 29-33.
